# Identification of SARS-CoV-2 and additional respiratory pathogens cases under the investigation of COVID-19 initial phase in a Brazilian reference laboratory

**DOI:** 10.1590/0074-02760200232

**Published:** 2020-09-18

**Authors:** Aline da Rocha Matos, Fernando Couto Motta, Braulia Costa Caetano, Maria Ogrzewalska, Cristiana Couto Garcia, Jonathan Christian Oliveira Lopes, Milene Miranda, Miriam Teresinha Furlam Prando Livorati, André Abreu, David Brown, Marilda Mendonça Siqueira

**Affiliations:** 1 Fundação Oswaldo Cruz-Fiocruz, Instituto Oswaldo Cruz, Laboratório de Vírus Respiratórios e do Sarampo, Rio de Janeiro, RJ, Brasil; 2 Ministério da Saúde, Secretaria de Vigilância em Saúde, Coordenação-Geral de Laboratórios, Brasília, DF, Brasil

**Keywords:** SARS-CoV-2, COVID-19, surveillance, laboratory

## Abstract

Coronavirus disease 2019 (COVID-19) surveillance, in Brazil, initiated shortly after its description, in China. Our aim was to detect severe acute respiratory syndrome coronavirus 2 (SARS-CoV-2) and additional pathogens in samples from the initial phase of the outbreak in Brazil, from late February to late March. From 707 samples analysed, 29 (4.1%) were SARS-CoV-2 positive. Fever and cough were their most prevalent symptoms. Co-detection of rhinovirus was observed in 2 (6.9%) cases. Additional pathogens were identified in 66.1% of the SARS-CoV-2 negative cases, mainly rhinovirus and influenza A(H1N1)pdm09. Thus, we emphasise the importance of differential diagnosis in COVID-19 suspected cases.

The notification of the first cases of infection with the severe acute respiratory syndrome coronavirus 2 (SARS-CoV-2) in Wuhan City, China, on 31 December 2019, was followed by a rapid and unprecedent virus spread worldwide.^1^ Currently, almost 13.4 million cases and 600,000 fatalities of SARS-CoV-2 disease [Coronavirus disease 2019 (COVID-19)] have been reported in the world.^2^ In Brazil, over 1,800,000 cases and 72,000 deaths have been identified so far.

Brazilian surveillance of SARS-CoV-2 cases began at the end of January, 2020. Ministry of Health (MoH) has defined the criteria for testing the suspected cases, which have been constantly updated.^3^ Initially, suspected cases were determined as symptomatic individuals, presenting with fever and other signs of respiratory infection, independent of severity, who had recently travelled to countries with SARS-CoV-2 community transmission. In the end of February, the first SARS-CoV-2 positive cases were detected in the country, in individuals who had a history of returning from affected countries. However, since March 20th, widespread national community transmission of COVID-19 has been recognised in Brazil. Sustained transmission was initially observed in the big metropolis, as São Paulo and Rio de Janeiro.

COVID-19 suspected samples were initially tested based on the network of laboratories established as part of the Influenza Surveillance System, which were already implemented in all Brazilian States. As the number of potential cases dramatically increased, additional laboratories were included as testing facilities. The Laboratory of Respiratory Viruses and Measles, at Fiocruz, Rio de Janeiro, is a National Influenza Centre for the Brazilian MoH and has been recently designated as one of the COVID-19 Reference Laboratories of the World Health Organization. Thus, we routinely receive samples from nine out of 27 Brazilian states (including Southern, Northeastern and Southern regions). Exceptionally, during this initial phase of the investigation, we also received samples from five additional states (from Midwestern, Northeastern and Northern regions), including from individuals who were quarantined upon their return from countries with SARS-CoV-2 communitarian transmission.

Over the course of this investigation, that was conducted from late February until late March, therefore the initial phase of the COVID-19 outbreak in Brazil, from the identification of the first cases and the statement of the communitarian transmission in the country, we have analysed 707 samples from COVID-19 suspected cases submitted to the influenza surveillance network. Respiratory specimens (mostly nasopharyngeal swabs) were subjected to total RNA extraction, by using commercial kits (QIAmp Viral RNA Mini kit - Qiagen, Germany), followed by SARS-CoV-2 detection by a TaqMan real time reverse-transcriptase polymerase chain reaction (RT-PCR) protocol targeting amplification of the Envelope and Polymerase genes, which had been previously described.^4^ The initial assays were performed with sets of primers and probes provided by Pan American Health Organization.^5^ Later on, viral detection was performed using a kit (based on the same targets) produced and validated by Bio-Manguinhos/Fiocruz, Brazil,^6^ which also included as the internal control human RNP. This protocol was further registered by the Brazilian regulatory agency ANVISA as registration number 25351.225181/2020-88. Further, all the SARS-CoV-2 positive samples were submitted to differential diagnosis, by using the XGEN multiplex platform to detect additional 21 respiratory pathogens (Mobius Life Science, Brazil). Moreover, 165 SARS-CoV-2 negative cases were tested for this multiplex platform in order to detect the etiological agent of the respiratory infection. Testing only a subset of the SARS-CoV-2 negative cases was limited due to the high cost of this assay.

As a result, during the period of our analysis, we have identified 29 (4.1%) SARS-CoV-2 positive cases. 58.6% of cases were male and the median age of the patients was 47 years old (ranging from 27 to 81). In relation to their symptoms, they presented with fever (measured or reported) (69.2%), cough (69.2%), sore throat (38.5%) and dyspnea (15.4%). Noteworthy, 22.2% of these individuals had preexisting chronic diseases and 54.5% were admitted to the hospital (Table I). Importantly, most of the cases, in the course of this analysis, were from individuals with recent travel abroad history. Noteworthy, roughly half of the total positive cases were collected from individuals from the Southeast region, which was the initial Brazilian COVID-19 epicentre,^7^ followed by the Midwest region, that comprised positive cases from Brasília from individuals that returned from an international trip. In our analysis, two (6.9%) of the 29 SARS-CoV-2 positive individuals also were positive for the detection of rhinovirus.

**TABLE I:** Clinical, geographical and epidemiological characteristics of the severe acute respiratory syndrome coronavirus 2 (SARS-CoV-2) positive cases received at the coronavirus disease 2019 (COVID-19) Brazilian reference laboratory, at Fiocruz

Characteristics	Total
Number of samples	29
Age (years) - median ± range	47 (27-81)
Male - n (%)	17 (58.6)
Signs and symptoms	
Fever - n/N (%)	9/13 (69.2)
Cough - n/N (%)	9/13 (69.2)
Sore throat - n/N (%)	5/13 (38.5)
Dyspnea - n/N (%)	2/13 (15.4)
Hospital admission - n/N (%)	6/11 (54.5)
Comorbidities - n/N (%)	2/9 (22.2)
Geographic region	
South - n (%)	3 (10.4)
Southeast - n (%)	13 (44.8)
Northeast - n (%)	4 (13.8)
Midwest - n (%)	9 (31.0)
North - n (%)	ND

ND: not detected; n: number of cases with the referred characteristic; N: number of cases for which the information is available.

Furthermore, in the 165 (23.3%) SARS-CoV-2 negative individuals screened for additional pathogens, at least one etiological agent was detected in 109 (66.1%) of the cases (Table II). Rhinovirus was the most prevalent pathogen, detected in 46 (27.9%) of the cases, followed by 18 (10.9%) cases of influenza A(H1N1)pdm09 and 12 (7.3%) cases of enterovirus. In these 165 SARS-CoV-2 negative cases, 41 (24.8%) presented with co-detection of more than one pathogen. Of note, two cases resulted in a fatality. One of them, a 60-year-old male, who presented with pneumopathy, cardiopathy, diabetes and was HIV-positive, had a rhinovirus infection. The other one, a 50-year-old female with no comorbidities reported, was positive for influenza A(H1N1)pdm09.

**TABLE II:** Identification of pathogens in severe acute respiratory syndrome coronavirus 2 (SARS-CoV-2) suspected patients received at the coronavirus disease 2019 (COVID-19) Brazilian reference laboratory, at Fiocruz

Pathogen	SARS-CoV-2 positive (n = 29)	SARS-CoV-2 negative (n = 165)
	n (%)	n (%)
Coronavirus NL63	ND	9 (5.5)
Coronavirus 229E	ND	4 (2.4)
Coronavirus OC43	ND	ND
Coronavirus HKU1	ND	7 (4.2)
Influenza A(H1N1)pdm09	ND	18 (10.9)
Influenza A	ND	11 (6.7)
Influenza B	ND	9 (5.5)
Parainfluenza 1	ND	2 (1.2)
Parainfluenza 2	ND	ND
Parainfluenza 3	ND	3 (1.8)
Parainfluenza 4	ND	ND
Human metapneumovirus A/B	ND	3 (1.8)
Rhinovirus	2 (6.9)	46 (27.9)
Respiratory sincicial virus	ND	5 (3.0)
Enterovirus	ND	12 (7.3)
Parechovirus	ND	ND
Adenovirus	ND	8 (4.8)
Bocavirus	ND	ND
Mycoplasma pneumoniae	ND	1 (0.6)

ND: not detected.

These results represent a description of the surveillance being conducted in a South American country for the identification of the SARS-CoV-2 cases. So far, most of the confirmed cases, in Brazil, have been mild cases. In this study, fever and cough were the most common symptoms of SARS-CoV-2 patients, which is consistent with a meta‐analysis which included data from 50,466 patients with SARS‐CoV‐2 infection.^8^

As in other countries, the number of cases is rising exponentially, and the availability of the diagnostic kits is a major limitation. Therefore, the MoH has recommended social isolation for all asymptomatic individuals that are non-key workers and the mild symptomatic cases of influenza like illness (ILI), regardless of the causative agent. Testing is currently mostly performed in the cases that present with symptoms, such as difficulty of breathing and high fever to support clinical management. Thus, although the identification of severe cases has already increased in the following weeks/months, the true total number of cases probably is dramatically underestimated.

It is important to highlight that, in Brazil, this study was conducted at the beginning of the influenza season, when other respiratory virus infections are also more prevalent.^9^ This represents a great challenge for COVID-19 surveillance. Further analysis over the following months will show how the emergence and co-circulation of SARS-CoV-2 will impact with the concomitant circulation of other seasonal respiratory viruses. Phylogeographical and phylogenetic studies are being conducted by others and us and will better elucidate the pattern of evolution and the chains of transmission of SARS-CoV-2 in the country.

During the period of our study, which occurred in the initial month of the early epidemic phase in Brazil, Southeast accounted for most of the Brazilian COVID-19 cases, mainly from São Paulo and Rio de Janeiro states.^7^ Since we do not receive samples from São Paulo due to the organisation of our surveillance system, most of our Southeastern cases were from Rio de Janeiro, the second most important COVID-19 epicenter in Brazil at that period.^7^ Midwest region was the second most prevalent in our cohort, due to the inclusion of positive cases that returned from an international trip. Northeastern and Southern cases were still low, which also represented the COVID-19 scenario at that moment. Finally, as we do not receive samples from the North region systematically, also because of the organisation of the surveillance network, they were underrepresented in our cohort.

Additionally, co-detection of an additional pathogen in the SARS-CoV-2 cases has already been described in a similar frequency to previous reports, in distinct populations.^10^ ^,^ ^11^ ^,^ ^12^ Our results described that 6.9% of the SARS-CoV-2 samples also have detection of an additional pathogen (rhinovirus). Consistently, rhinovirus was also the most prevalent pathogen detected in our SARS-CoV-2 negative cases. Additional comprehensive analysis will determine the importance of the co-detection for the clinical presentation and evolution of cases, as well as for the future construction of the test algorithms.

Our report emphasises the importance of differential diagnosis in suspected cases of COVID-19, because many of its symptoms are similar to those found in other common respiratory viral infection. The identification of the specific pathogen infecting the individual is important for public health and may contribute to treatment, when there is an available therapy, as in the case of influenza. Furthermore, the differentiation of the pathogens presenting with a similar respiratory syndrome has the potential to limit unnecessary quarantine/isolation, especially for key workers, although it is very difficult and expensive to implement for the entire population. This may be important, if we consider the burden that mass quarantine imposes on essential services, such as health, safety and food production, due to withdraw of workers from their activities.
